# Comparison of Dose Distribution in Regional Lymph Nodes in Whole-Breast Radiotherapy vs. Whole-Breast Plus Regional Lymph Node Irradiation: An In Silico Planning Study in Participating Institutions of the Phase III Randomized Trial (KROG 1701)

**DOI:** 10.3390/cancers12113261

**Published:** 2020-11-04

**Authors:** Haeyoung Kim, Heejung Kim, Won Park, Jong Yun Baek, Sung Ja Ahn, Mi Young Kim, Shin-Hyung Park, Ik Jae Lee, Inbong Ha, Jin Hee Kim, Tae Hyun Kim, Kyu Chan Lee, Hyung-Sik Lee, Tae Gyu Kim, Jin Ho Kim, Jong Hoon Lee, Jinhong Jung, Oyeon Cho, Jee Suk Chang, Eun Seog Kim, In Young Jo, Taeryool Koo, Kyubo Kim, Hae Jin Park, Young-Joo Shin, Boram Ha, Jeanny Kwon, Ju Hye Lee, Sunrock Moon

**Affiliations:** 1 Department of Radiation Oncology, Samsung Medical Center, Sungkyunkwan University School of Medicine, Seoul 06351, Korea; haeyoung0131.kim@samsung.com (H.K.); heejung0228.kim@samsung.com (H.K.); jy2.baek@samsung.com (J.Y.B.); 2 Department of Radiation Oncology, Chonnam National University Medical School, Gwangju 58128, Korea; ahnsja@chonnam.ac.kr; 3 Department of Radiation Oncology, Kyungpook National University Chilgok Hospital, Daegu 41404, Korea; cucici912@gmail.com; 4 Department of Radiation Oncology, Kyungpook National University School of Medicine, Daegu 41944, Korea; shinhyungpark@knu.ac.kr; 5 Department of Radiation Oncology, Gangnam Severance Hospital, Yonsei University College of Medicine, Seoul 06273, Korea; ikjae412@yuhs.ac; 6 Department of Radiation Oncology, Gyengsang National University Hospital, Gyengsang National University School of Medicine, Jinju 52727, Korea; nicehib@gnuh.co.kr; 7 Department of Radiation Oncology, Dongsan Medical Center, Keimyung University School of Medicine, Daegu 41931, Korea; jhkim@dsmc.or.kr; 8 National Cancer Center, Research Institute and Hospital, Center for Proton Therapy, Goyang 10408, Korea; k2onco@ncc.re.kr; 9 Department of Radiation Oncology, Gil Medical Center, Gachon University College of Medicine, Incheon 21565, Korea; kyu22@gilhospital.com; 10 Department of Radiation Oncology, Dong-A University Hospital, Dong-A University School of Medicine, Busan 49201, Korea; hyslee@dau.ac.kr; 11 Department of Radiation Oncology, Samsung Changwon Hospital, Sungkyunkwan University School of Medicine, Changwon 51353, Korea; ktg7757@hanmail.net; 12 Department of Radiation Oncology, Seoul National University Hospital, Seoul 03080, Korea; jinhokim@snuh.org; 13 Department of Radiation Oncology, St. Vincent’s Hospital, The Catholic University of Korea, Suwon 16247, Korea; koppul@catholic.ac.kr; 14 Department of Radiation Oncology, Asan Medical Center, University of Ulsan College of Medicine, Seoul 05505, Korea; jung.jinhong@amc.seoul.kr; 15 Department of Radiation Oncology, Ajou University School of Medicine, Suwon 16499, Korea; oyeoncho@aumc.ac.kr; 16 Department of Radiation Oncology, Yonsei Cancer Center, Yonsei University College of Medicine, Seoul 03722, Korea; changjeesuk@yuhs.ac; 17 Department of Radiation Oncology, Soonchunhyang University Hospital, Soonchunhyang University College of Medicine, Cheonan 31151, Korea; radio@schmc.ac.kr (E.S.K.); inyoung.jo@schmc.ac.kr (I.Y.J.); 18 Department of Radiation Oncology, Hallym University Sacred Heart Hospital, Hallym University College of Medicine, Anyang 14068, Korea; kootaeryool@hallym.or.kr; 19 Department of Radiation Oncology, Ewha Womans University College of Medicine, Seoul 07804, Korea; kyubokim@ewha.ac.kr; 20 Department of Radiation Oncology, Hanyang University College of Medicine, Seoul 15588, Korea; haejinpark@hanyang.ac.kr; 21 Department of Radiation Oncology, Inje University Sanggye Paik Hospital, Seoul 07804, Korea; fluty99@empal.com; 22 Department of Radiation Oncology, Hallylm University Dongtan Sacred Heart Hospital, Hwaseong 14068, Korea; boramhaa@hallym.or.kr; 23 Department of Radiation Oncology, Chungnam National University Hospital, Daejeon 35015, Korea; ijeannyi@daum.net; 24 Department of Radiation Oncology, Pusan National University Yangsan Hospital, Yangsan 50612, Korea; gg220110@goodhospital.or.kr; 25 Department of Radiation Oncology, Wonkwang University Hospital, Wonkwang University School of Medicine, Iksan 54538, Korea; sunrmoon@wku.ac.kr

**Keywords:** breast cancer, lymph nodes, radiotherapy planning, randomized trial

## Abstract

**Simple Summary:**

The purpose of the current in silico planning study is to compare radiation doses of whole-breast irradiation (WBI) and whole-breast plus regional lymph node irradiation (WBI+RNI) administered to the regional lymph nodes (RLN) in pN1 breast cancer. Twenty-four participating institutions were asked to create plans of WBI and WBI+RNI for two dummy cases. In all RLN regions including supraclavicular lymph node, axillary lymph node, and internal mammary lymph node, the radiation dose to the RLN was higher in WBI+RNI plan than WBI plan.

**Abstract:**

The purpose of the current in silico planning study is to compare radiation doses of whole-breast irradiation (WBI) and whole-breast plus regional lymph node irradiation (WBI+RNI) administered to the regional lymph nodes (RLN) in pN1 breast cancer. Twenty-four participating institutions were asked to create plans of WBI and WBI+RNI for two dummy cases. To compare target coverage between the participants, an isodose line equal to 90% of the prescribed dose was converted to an isodose contour (contour_90% iso_)_._ The relative nodal dose (RND) was obtained using the ratio of RLN dose to the target dose. The Fleiss’s kappa values which represent inter-observer agreement of contour_90% iso_ were over 0.68. For RNI, 6 institutions included axillary lymph node (ALN), supraclavicular lymph node (SCN), and internal mammary lymph node (IMN), while 18 hospitals included only ALN and SCN. The median RND between the WBI and WBI+RNI were as follows: 0.64 vs. 1.05 (ALN level I), 0.27 vs. 1.08 (ALN level II), 0.02 vs. 1.12 (ALN level III), 0.01 vs. 1.12 (SCN), and 0.54 vs. 0.82 (IMN). In all nodal regions, the RND was significantly lower in WBI than in WBI+RNI (*p* < 0.01). In this study, we could identify the nodal dose difference between WBI and WBI+RNI.

## 1. Introduction

Whole-breast irradiation (WBI) after breast-conserving surgery (BCS) reduces locoregional recurrence and improves survival in patients with breast cancer [1]. To eradicate microscopic regional disease, prophylactic regional lymph node irradiation (RNI) is administered along with WBI in early-stage breast cancer. Including RNI was expected to prevent systemic spreading of cancer and to improve the survival of the patient [2]. RNI is regarded as a standard treatment in patients with four or more axillary lymph node (ALN) metastasis [3]. However, in cases with 1 to 3 positive ALN (pN1), it remains uncertain whether RNI has a beneficial impact on the survival of the patient [4]. In previous randomized trials, RNI was significantly associated with improved disease-free survival (DFS) in patients with high-risk node-negative or pN1 breast cancer [5,6]. However, in these studies, only a small proportion of patients received contemporary systemic treatments such as taxane or anti-HER2 agents that have been proven to improve locoregional control. It is possible that the benefit of RNI found in the studies was caused by adopting a less effective systemic treatment. Therefore, it is necessary to evaluate the prognostic impact of RNI in pN1 breast cancer patients receiving contemporary systemic treatments.

A phase III randomized trial was initiated by the Korean Radiation Oncology Group (the KROG 1701 study, NCT03269981) to analyze the impact of RNI in pN1 breast cancer patients receiving effective systemic therapies [7]. The primary objective of the KROG 1701 study was to compare DFS between WBI and WBI plus RNI (WBI+RNI) in pN1 breast cancer patients who received BCS and taxane-based chemotherapy. For adequate interpretation of the KROG 1701 study result, it is necessary to ensure different radiation dose to the regional lymph nodes (RLN) between WBI and WBI+RNI. We performed this in silico study to compare radiation dose distribution in the RLN between WBI and WBI+RNI plans among participating institutions of the KROG 1701 study.

## 2. Results

### 2.1. General Information

In this planning study, 22 institutions used three-dimensional conformal radiotherapy (3D-CRT) while 2 hospitals used intensity-modulated radiotherapy (IMRT). For RLN radiotherapy in the WBI+RNI arm, 6 hospitals included ALN, supraclavicular lymph node (SCN), and internal mammary lymph node (IMN), while 18 institutions included only ALN and SCN in the treatment volumes. In each institution, the same fractionation schedule with once-daily radiotherapy was applied for both treatment arms. The prescription schemes were as follows: a total dose of 50.0 Gy in 25 fractions (*n* = 10), 50.4 Gy in 28 fractions (*n* = 8), 43.2 Gy in 16 fractions (*n* = 2), 41.6 Gy in 16 fractions (*n* = 1), 40.5 Gy in 15 fractions (*n* = 1), 40.05 Gy in 15 fractions (*n* = 1), and 40.0 Gy in 16 fractions (*n* = 1).

**Figure 1:**
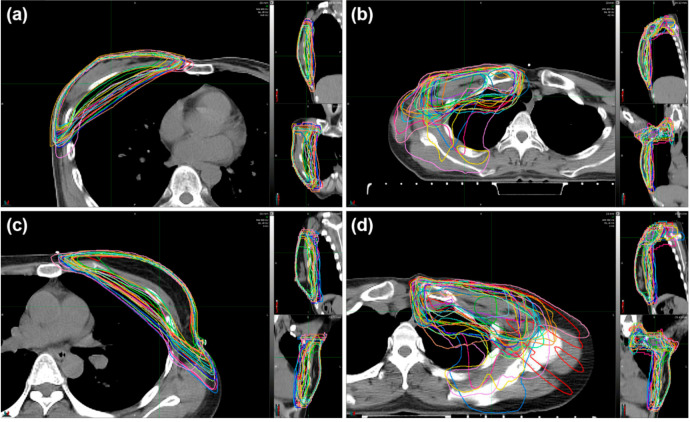
Isodose contours representing 90% of the prescribed dose in each institution for the cases of (**a**) WBI arm in Case A, (**b**) WBI+RNI arm in Case A, (**c**) WBI arm in Case S, and (**d**) WBI+RNI arm in Case S. Abbreviations: WBI, whole-breast irradiation; WBI+RNI, whole-breast irradiation plus regional nodal irradiation.

Distributions of the 24 contour_90% iso_ in both treatment arms are depicted in Figure 1; WBI arm in Case A (a), WBI+RNI arm in Case A (b), WBI arm in Case S (c), and WBI+RNI arm in Case S (d). 

The Fleiss’s kappa values among 24 contour_90% iso_ in all four situations (WBI in Case A, WBI+RNI in Case A, WBI in Case S, and WBI+RNI in Case S) were over 0.68. The DSC value was calculated between each institutional contour_90% iso_ and the Ref-contour_90% iso_. The median DSC value of the 24 institutions for the four situations was as follows: 0.80 for WBI arm in Case A, 0.77 for WBI+RNI arm in Case A, 0.83 for WBI arm in Case S, and 0.80 for WBI+RNI arm in Case S (Table 1).

For the WBI plan, the DSC values were over 0.68 in all institutions except for one. For the WBI+RNI plan, the DSC values were over 0.68 in all institutions except for four (Figure S1).

### 2.2. Comparisons of RLN and OAR Dose between the Treatment Arms

**Figure 2:**
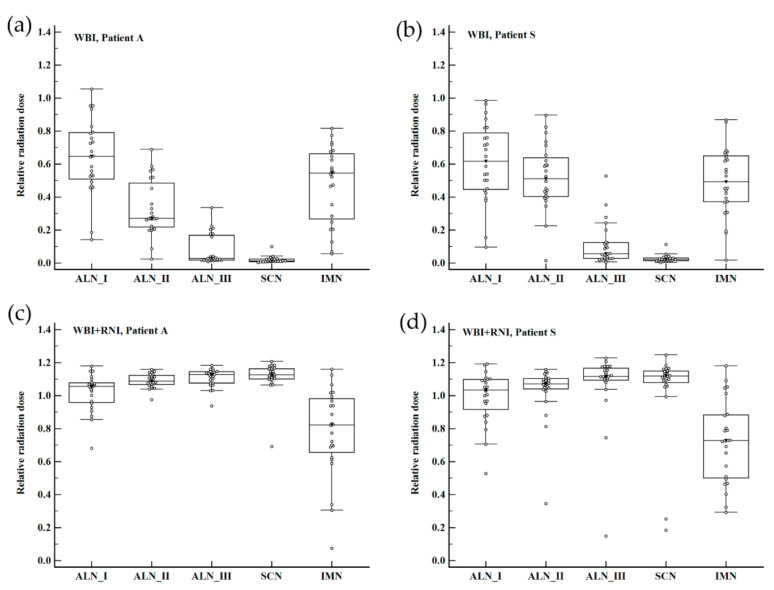
Box-and-whisker plots for relative nodal radiation dose according to the regional node group and treatment arms among 24 participating institutions. (**a**) WBI in Case A, (**b**) WBI in Case S, (**c**) WBI+RNI in Case A, and (**d**) WBI+RNI in Case S. Abbreviation: WBI, whole-breast irradiation; WBI+RNI, whole-breast irradiation plus regional nodal irradiation; ALN I, axillary node level I; ALN II, axillary node level II; ALN III, axillary node level III; SCN, supraclavicular lymph node; IMN, internal mammary lymph node.

In both WBI and WBI+RNI plans, all regions of RLN received various amounts of radiation. The median values of relative mean radiation dose to RLN ranged between 0.01 and 0.64 for WBI plans and between 0.72 and 1.12 for WBI+RNI plans. WBI in Case A (a), WBI in Case S (b), WBI+RNI in Case A (c), and WBI+RNI in Case S (d) are depicted in Figure 2.

Relative IMN radiation dose was different depending on whether the IMN was included in the treatment volume or not. The median value of the relative radiation dose for IMN was 1.04 in WBI+RNI plans that included IMN irradiation. In WBI+RNI plans without IMN irradiation, the median relative IMN radiation dose ranged between 0.07 and 1.02 (Table S1). Regardless of RLN groups, the relative RLN radiation dose was significantly lower in the WBI treatment arm than in the WBI+RNI arm (Table 2).

Ipsilateral lung dose was significantly higher in the WBI+RNI than the WBI arm. All values of lung dosimetric parameters were higher in the WBI+RNI than in the WBI arm. Mean heart dose ranged between 0.1 Gy and 10.6 Gy in all four plans of Case A and Case S. There was no significant difference in mean heart dose between the two treatment arms (Table 3).

However, in the WBI+RNI plan for Case A, the median value of mean heart dose was significantly higher among institutions that included IMN than those that did not (0.5 Gy vs. 4.7 Gy, *p* < 0.01). However, the difference in mean heart dose depending on IMN irradiation was not significant in Case S (Table S2).

## 3. Discussion

In this in silico study, we found moderate-to-strong agreement on radiotherapy treatment volume between the participants for both Case A and Case S. In an evaluation of relative radiation dose for RLN in both treatment arms, we found that significant proportions of radiation were unintentionally delivered to ALN level I and IMN in the WBI arm. Nonetheless, relative radiation doses for all RLN regions were significantly higher in the WBI+RNI arm than in the WBI arm. All dosimetric parameters of the ipsilateral lung were higher in the WBI+RNI arm than in the WBI arm. However, the mean radiation dose for the heart was not significantly different in the two treatment arms.

Prophylactic irradiation of SCN and IMN was provided for patients with pN1 breast cancer to eliminate microscopic tumor foci in RLN regions [8]. Although previous randomized trials such as the MA.20 and the EORTC 22922-10925 have shown the benefit of RNI in breast cancer control, these two studies accrued patients before 2007 when effective modern systemic agents were not easily available [5,6]. Therefore, the magnitude of the RNI benefit is unclear in pN1 breast cancer patients who underwent modern systemic therapies. Thus, there was a need to reevaluate the impact of RNI that reflected the effects of contemporary systemic agents. In a recent study assessing the impact of RNI among node-positive HER2-positive breast cancer patients who were enrolled in the Adjuvant Lapatinib And/Or Trastuzumab Treatment Optimisation (ALTTO) trial [9], there was no significant association between RNI and DFS in patients receiving optimal anti-HER2 targeted therapies [10]. In a similar vein, the phase III KROG 1701 study has been underway with the aim to compare DFS between WBI and WBI+RNI in patients receiving taxane-based chemotherapy and molecular subtype-specific treatment, including endocrine therapy or anti-HER2 therapies. In the previous trials, the extents of RNI varied among the studies. RNI was performed, including both SCN and IMN in the MA.20 and EORTC 22922-10925 trials; SCN and/or IMN was covered in RNI among patients participating in the ALTTO trial. Because the extent of RNI might have an influence on DFS, it is necessary to take account of the RNI extent in the evaluation of the trial outcomes. Moreover, the quality of radiotherapy among the participating institutions should be assured to analyze the impact of RNI accurately.

In this study, the 24 participating institutions of the KROG 1701 study were requested to create radiotherapy plans for two cases. The two cases were set up to represent each side of breast cancer and the two methods for ALN management: Case A had ALND for right breast cancer, while Case S received SLNBx for left breast cancer. By comparing each institutional contour_90% iso_ for the two cases, we aimed to evaluate patterns of radiotherapy planning and agreement of dose coverage among the participants. In other dummy run studies of phase III randomized trials on RNI for breast cancer, a comparison of IMN radiation dose between the treatment arms was the main focus of interest [11,12]. Unlike these studies, we compared the radiation dose for the whole breast and all groups of RLN depending on treatment arms among the participating institutions. The Fleiss’s kappa values were over 0.68 among 24 contour_90% iso_ in all four situations. Further, values of DSC between the Ref-contour_90% iso_ and each institutional contour_90% iso_ were over 0.68 in all but two institutions. In these two institutions, the DSC value was under 0.6 in both plans for WBI and WBI+RNI.

By comparing the nodal radiation dose between the arms, we found that all nodal groups received a larger radiation dose in the WBI+RNI arm than in the WBI arm. The RND tends to be larger than 1 in most of the WBI+RNI plans. Three-field techniques, which were used for WBI+RNI, frequently result in an overdose area in the filed junction. The overdose areas are probably attributable to the RND ≥ 1 in our study. The RLN radiation dose was different depending on the extent of the radiotherapy field. In WBI+RNI, the relative nodal dose in IMN differed based on whether IMN was included in the treatment volume or not. Moreover, the radiation dose to ALN level I differed based on the use of IMN irradiation. Thus, the dosimetric effect of IMN radiotherapy should be considered while interpreting the outcomes of the KROG 1701 study in the future. Further, we found in this study that IMN receives 5–80% of the prescription dose even in WBI. In the dummy run of the EORTC 22922-10925 trial, radiation dose in the IMN was less than 50% of the prescription dose in over 94% of the plans of the WBI group [11]. Another dummy run study of a randomized trial on IMN irradiation (KROG 08-06 study) reported that mean radiation dose to IMN was 40–74% of the prescription dose in the WBI arm [12]. The dummy run of the EORTC 22922-10925 trial evaluated point dose rather than a volumetric dose of the IMN. Additionally, radiation dose distributions were checked in three slices of simulation computed tomography (CT) and not all slices of CT [11]. Therefore, the radiation dose to IMN might be over- or under-estimated in the dummy run of the EORTC 22922-10925 trial. In the dummy run of the KROG 08-06 study [12], the range of IMN radiation dose in WBI was smaller than that in our study. The dummy run of the KROG 08-06 study was performed in 12 institutions, whereas the current planning study was conducted in 24 institutions. It is probable that due to the larger number of participating institutions in our study, the range of IMN radiation dose was larger than that in the dummy run of the KROG 08-06 trial.

We found that all dosimetric parameters of the ipsilateral lung were higher in the WBI+RNI than in the WBI arm. This can be related to lung toxicity. In a previous study on prophylactic SCN irradiation in pN1 breast cancer, a higher frequency of grade I pneumonitis was observed in the case of SCN radiotherapy with WBI than in WBI alone [13]. Even though lung toxicity might not be severe after WBI+RNI, the radiotherapy plan should be modified to minimize lung dose. We recognized that the mean heart dose was not significantly different between the two arms in this planning study. Among the KROG 1701 study participants, only a quarter of the institutions included IMN in the treatment field for WBI+RNI plan. The mean heart dose was higher in institutions performing IMN-including RNI than that in those performing IMN-excluding RNI. However, statistical significance was noted only in Case A and not in Case S. The location of IMN and the patient’s geometric characteristics might have contributed to the difference in statistical significance between Case A and Case S. The range of mean heart dose was found to be large in this study. Since the mean heart dose in the contemporary radiotherapy era was reported to be <5 Gy [14,15], the mean heart dose noted in some institutions among our participants is thought to be high. The relative risk of major coronary events increases linearly with increasing mean heart dose (7.4% per Gy) [14]. Therefore, heart dose should be minimized whenever possible. In addition, we recognized that further efforts including education and feedback are needed to enhance homogeneity of radiation planning among participating institutions.

## 4. Materials and Methods

### 4.1. Cases

Investigators of the 24 participating institutions were asked to generate radiotherapy plans for two cases with pN1 breast cancer. Planning computed tomography (CT) images of two de-identified patients were used for this planning study. Details of the two cases are described in Table 4.

Briefly, one case (Case A) is of a patient with right breast cancer who received sentinel lymph node biopsy (SLNBx) followed by axillary lymph node dissection (ALND) and the second case (Case S) is of a patient with left breast cancer who underwent SLNBx only. CT images of the cases were downloaded from a website by the participants and loaded into each institutional treatment planning system. According to the radiotherapy guidelines of the KROG 1701 study (Table S3), each participant generated radiotherapy plans for both treatment arms, WBI and WBI+RNI, for the two cases. Thus, a total of four plans were created by each participating institution. Then, the participants were asked to upload the planning CT, structure sets, and radiotherapy plans to a website in the Digital Imaging and Communications in Medicine format. Planning for tumor bed boost was not required in this study. The study was approved by the Institutional Review Board of Samsung Medical Center, SMC 2017-01-085-001.

### 4.2. Data Analysis

To analyze the target dose coverage, an isodose line equal to 90% of the prescribed dose was extracted from the radiotherapy plan of each participating institution. Then, the isodose line was converted to an isodose contour (contour_90% iso_)_._ An agreement of contour_90% iso_ among the participating institutions was assessed by the Computational Environment for Radiotherapy Research software [16]. Further, based on the contour_90% iso_ of 24 participating institutions, a reference isodose contour (Ref-contour_90% iso_) was created by the simultaneous truth and performance level estimation (STAPLE) algorithm (Figure S2) [17]. The isodose contour of WBI in Case A (a), WBI+RNI in Case A (b), WBI in Case S (c), and WBI+RNI in Case S (d) are presented in Figure S2. 

A similarity between each institutional contour_90% iso_ and the Ref-contour_90% iso_ was evaluated by calculating Dice similarity coefficient (DSC) using the 3D Slicer software [18]. The value of a DSC ranges from 0, which refers to an absence of spatial overlap between two samples, to 1, which refers to complete overlap. To assess radiation dose to the RLN according to treatment arms, a relative mean nodal radiation dose of each RLN was acquired. The relative nodal dose is defined as the ratio of RLN dose to the target prescription dose. RLN groups including ALN, supraclavicular lymph node (SCN), and/or internal mammary lymph node (IMN) were delineated according to the ESTRO (European Society for Radiotherapy and Oncology) consensus guideline [19] by two radiation oncologists (H. Kim and J.Y. Baek).

Using dose–volume histograms for the heart and lungs, dosimetric parameters of organs-at-risk (OAR) were evaluated. Mean lung and heart doses and lung volumes receiving more than 5 Gy (V_5 Gy_), 10 Gy (V_10 Gy_), 20 Gy (V_20 Gy_), and 30 Gy (V_30 Gy_) were analyzed. Because various fractionation schemes were used among participating institutions, radiation dose was converted to the biologically equivalent dose in 2 Gy fractions (EQD2) assuming the α/β ratio of 3 Gy for OAR [20].

### 4.3. Statistical Analysis

An agreement of contour_90% iso_ among the participating institutions was assessed using Fleiss’s Kappa method [21]. The Kappa value ranges between 0, which refers to no agreement, and 1, which refers to perfect agreement [22]. The Wilcoxon signed-rank test was performed to evaluate differences in RLN and OAR radiation doses between the two treatment arms. Statistical analyses were conducted using MedCalc Statistical Software version 18.11.3 (MedCalc Software bvba, Ostend, Belgium), and *p*-values < 0.05 were considered statistically significant.

## 5. Conclusions

In this study, we found that radiation doses for all nodal regions were significantly higher in the WBI+RNI than in the WBI. Through this in silico planning study, we could identify the nodal dose difference between the two treatment arms of the KROG 1701 study.

## Figures and Tables

**Table 1:** Comparisons of dose coverage among 24 participating institutions.

		Contour_90% iso_ *		Dice Similarity Coefficient ^†^
Case		Median Volume (Range)	Kappa	Median (Range)
Case A	WBI	511 cc (307–721 cc)	0.77	0.80 (0.51–0.89)
	WBI+RNI	766 cc (469–1184 cc)	0.68	0.77 (0.58–0.90)
Case S	WBI	745 cc (418–1211 cc)	0.78	0.83 (0.71–0.94)
	WBI+RNI	1048 cc (468–1557 cc)	0.71	0.80 (0.68–0.89)

* An isodose line equal to 90% of the prescribed dose was extracted from the radiotherapy plan of each participating institution. Then, the isodose line was converted to an isodose contour (contour_90% iso_). ^†^ Based on contour_90% iso_ of 24 participating institutions, a reference isodose contour (Ref-contour_90% iso_) was created. Dice similarity coefficient between each institutional contour_90% iso_ and the Ref-contour_90% iso_ was calculated. Abbreviations: WBI, whole-breast irradiation; WBI+RNI, whole-breast irradiation plus regional nodal irradiation; cc, cubic centimeter.

**Table 2:** Comparisons of relative mean nodal radiation dose between the treatment arms.

	Case A (Rt, ALND)			Case S (Lt, SLNBx)		
Regions	WBI	WBI+RNI	*p*-Value	WBI	WBI+RNI	*p*-Value
Axillary level I	0.64 (0.14–1.05)	1.05 (0.68–1.17)	<0.01	0.61 (0.09–0.98)	1.03 (0.52–1.19)	<0.01
Axillary level II	0.27 (0.02–0.68)	1.08 (0.97–1.15)	<0.01	0.51 (0.01–0.89)	1.07 (0.34–1.15)	<0.01
Axillary level III	0.02 (0.01–0.33)	1.12 (0.93–1.18)	<0.01	0.05 (0.00–0.52)	1.11 (0.14–1.22)	<0.01
SCN	0.01 (0.00–0.09)	1.12 (0.69–1.20)	<0.01	0.02 (0.00–0.11)	1.11 (0.18–1.24)	<0.01
IMN	0.54 (0.05–0.81)	0.82 (0.02–1.15)	<0.01	0.49 (0.01–0.86)	0.72 (0.29–1.18)	<0.01

The median value of mean relative radiation dose among 24 institutions was presented by nodal groups. The relative nodal dose is defined as the ratio of nodal dose to the target prescription dose. Values in parentheses present the range of relative nodal dose for each nodal group. Case A represents a patient with right breast cancer who received axillary lymph node dissection. Case S is a patient with left breast cancer who underwent sentinel lymph node biopsy. Abbreviations: Rt, right; ALND, axillary lymph node dissection; Lt, left; SLNBx, sentinel lymph node biopsy; WBI, whole-breast irradiation; WBI+RNI, whole-breast irradiation plus regional nodal irradiation; SCN, supraclavicular lymph node; IMN, internal mammary lymph node.

**Table 3:** Dosimetric parameters for lung and heart.

	Case A (Rt. Breast, ALND)			Case S (Lt. Breast, SLNBx)		
Parameters	WBI	WBI+RNI	*p*-Value	WBI	WBI+RNI	*p*-Value
Ipsilateral lung						
V_5 Gy_ (%)	30.2 (15.1–77.6)	46.8 (31.6–99.7)	<0.01	28.3 (11.8–81.5)	48.1 (27.8–99.9)	<0.01
V_10 Gy_ (%)	20.5 (10.1–44.1)	34.9 (19.2–97.4)	<0.01	20.7 (7.4–57.8)	36.7 (17.5–99.9)	<0.01
V_20 Gy_ (%)	14.3 (6.1–26.1)	28.2 (7.1–76.5)	<0.01	16.3 (3.9–29.0)	28.8 (7.2–74.0)	<0.01
V_30 Gy_ (%)	11.6 (2.1–20.5)	20.1 (1.5–38.4)	<0.01	12.9 (1.1–23.1)	20.2 (2.7–48.6)	<0.01
Mean dose (Gy)	8.8 (4.7–15.3)	13.9 (5.6–27.2)	<0.01	8.3 (3.0–16.8)	14.2 (5.4–29.3)	<0.01
Heart						
Mean dose (Gy)	0.5 (0.1–5.0)	0.9 (0.3–6.8)	0.07	3.7 (0.8–8.9)	4.4 (1.5–10.6)	0.09

Value in parentheses presents range of the value in each parameter. V_x Gy_ indicates percentage of lung volumes receiving more than x Gy. Case A represents a patient with right breast cancer who received axillary lymph node dissection. Case S is a patient with left breast cancer who underwent sentinel lymph node biopsy. Abbreviations: Rt, right; ALND, axillary lymph node dissection; Lt, left; SLNBx, sentinel lymph node biopsy; WBI, whole-breast irradiation; WBI+RNI, whole-breast irradiation plus regional nodal irradiation.

**Table 4:** Details of the cases.

Case	Description
Case A	43 years old. She received partial mastectomy and sentinel lymph node biopsy for right breast cancer. Intraoperative frozen sections of sentinel lymph nodes revealed metastatic carcinoma in 2 out of 3 sentinel lymph nodes. Complete axillary lymph node dissection was performed. The pathology showed a 2.5-cm-sized invasive ductal carcinoma of the breast. In the specimen of axillary lymph node dissection, there was no metastatic carcinoma in the lymph nodes.
Case S	40 years old. She received partial mastectomy and sentinel lymph node biopsy for cT1N0 cancer in the left breast. Intraoperative frozen sections of sentinel lymph nodes revealed metastatic carcinoma in 1 out of 4 sentinel lymph nodes. Axillary lymph node dissection was not performed. Pathology showed a 1.2-cm-sized invasive ductal carcinoma of the breast.

## Supplementary Materials

The following are available online at https://www.mdpi.com/2072-6694/12/11/3261/s1, Figure S1: Box-and-whisker plots for dice similarity coefficient among 24 participating institutions according to treatment arms, Figure S2: A reference 90% isodose contour that was created based on 90% isodose contours of 24 institutions using the STAPLE algorithm, Table S1: Relative nodal radiation dose according to the internal mammary lymph node irradiation in WBI+RNI arm, Table S2: Radiation dose to lung and heart depending on internal mammary lymph node irradiation in WBI+RNI arm, Table S3: Guidelines for radiation treatment in the KROG 1701 study.

## References

[1] Darby S., McGale P., Correa C., Taylor C., Arriagada R., Clarke M., Cutter D., Davies C., Ewertz M., Early Breast Cancer Trialists’ Collaborative Group (2011). Effect of radiotherapy after breast-conserving surgery on 10-year recurrence and 15-year breast cancer death: Meta-analysis of individual patient data for 10, 801 women in 17 randomised trials. Lancet.

[2] Poortmans P.M.P., Coles C., Bernier J. (2016). Treatment of Regional Lymph Nodes in Breast Cancer—Evidence in Favor of Radiation TherapyTreatment of Regional Lymph Nodes in Breast CancerTreatment of Regional Lymph Nodes in Breast Cancer. JAMA Oncol..

[3] The National Comprehensive Cancer Network NCCN Clinical Practice Guidelines in Oncology, Breast Cancer, Version 1.2019. https://www.nccn.org/professionals/physician_gls/pdf/breast.pdf..

[4] Harris J.R. (2016). Treatment of Regional Lymph Nodes in Breast Cancer—Not Recommended for All Patients With 1 to 3 Positive Auxiliary Nodes. JAMA Oncol..

[5] Poortmans P.M., Collette S., Kirkove C., Van Limbergen E., Budach V., Struikmans H., Collette L., Fourquet A., Maingon P., Valli M. (2015). Internal Mammary and Medial Supraclavicular Irradiation in Breast Cancer. N. Engl. J. Med..

[6] Whelan T.J., Olivotto I.A., Parulekar W.R., Ackerman I., Chua B.H., Nabid A., Vallis K.A., White J.R., Rousseau P., Fortin A. (2015). Regional Nodal Irradiation in Early-Stage Breast Cancer. N. Engl. J. Med..

[7] Kim H., Park W., Choi D., Ahn S., Kim S., Kim E., Lee J., Lee K., Kim J., Lee H.-S. Abstract OT2-04-02: A phase 3 study of post-lumpectomy radiotherapy to whole breast + regional lymph nodes vs whole breast alone for patients with pN1 breast cancer treated with taxane-based chemotherapy (KROG 1701): Trial in progress. Proceedings of the 2018 San Antonio Breast Cancer Symposium.

[8] Xie L., Higginson D.S., Marks L.B. (2011). Elective regional nodal irradiation in patients with early-stage breast cancer. Semin. Radiat. Oncol..

[9] Piccart-Gebhart M., Holmes E., Baselga J., de Azambuja E., Dueck A.C., Viale G., Zujewski J.A., Goldhirsch A., Armour A., Pritchard K.I. (2016). Adjuvant Lapatinib and Trastuzumab for Early Human Epidermal Growth Factor Receptor 2-Positive Breast Cancer: Results From the Randomized Phase III Adjuvant Lapatinib and/or Trastuzumab Treatment Optimization Trial. J. Clin. Oncol..

[10] Gingras I., Holmes E., De Azambuja E., Nguyen D.H., Izquierdo M., Anne Zujewski J., Inbar M., Naume B., Tomasello G., Gralow J.R. (2017). Regional Nodal Irradiation After Breast Conserving Surgery for Early HER2-Positive Breast Cancer: Results of a Subanalysis From the ALTTO Trial. J. Natl. Cancer Inst..

[11] Poortmans P.M., Venselaar J.L., Struikmans H., Hurkmans C.W., Davis J.B., Huyskens D., van Tienhoven G., Vlaun V., Lagendijk J.J., Mijnheer B.J. (2001). The potential impact of treatment variations on the results of radiotherapy of the internal mammary lymph node chain: A quality-assurance report on the dummy run of EORTC Phase III randomized trial 22922/10925 in Stage I–III breast cancer(1). Int. J. Radiat. Oncol. Biol. Phys..

[12] Chung Y., Kim J.W., Shin K.H., Kim S.S., Ahn S.J., Park W., Lee H.S., Kim D.W., Lee K.C., Suh H.S. (2015). Dummy run of quality assurance program in a phase 3 randomized trial investigating the role of internal mammary lymph node irradiation in breast cancer patients: Korean Radiation Oncology Group 08-06 study. Int. J. Radiat. Oncol. Biol. Phys..

[13] Kim H., Park W., Yu J.I., Choi D.H., Huh S.J., Kim Y.J., Lee E.S., Lee K.S., Kang H.S., Park I.H. (2017). Prognostic Impact of Elective Supraclavicular Nodal Irradiation for Patients with N1 Breast Cancer after Lumpectomy and Anthracycline Plus Taxane-Based Chemotherapy (KROG 1418): A Multicenter Case-Controlled Study. Cancer Res. Treat..

[14] Taylor C., Correa C., Duane F.K., Aznar M.C., Anderson S.J., Bergh J., Dodwell D., Ewertz M., Gray R., Jagsi R. (2017). Estimating the Risks of Breast Cancer Radiotherapy: Evidence From Modern Radiation Doses to the Lungs and Heart and From Previous Randomized Trials. J. Clin. Oncol..

[15] Dess R.T., Liss A.L., Griffith K.A., Marsh R.B., Moran J.M., Mayo C., Koelling T.M., Jagsi R., Hayman J.A., Pierce L.J. (2017). Ischemic Cardiac Events Following Treatment of the Internal Mammary Nodal Region Using Contemporary Radiation Planning Techniques. Int. J. Radiat. Oncol. Biol. Phys..

[16] Deasy J.O., Blanco A.I., Clark V.H. (2003). CERR: A computational environment for radiotherapy research. Med. Phys..

[17] Allozi R., Li X.A., White J., Apte A., Tai A., Michalski J.M., Bosch W.R., El Naqa I. (2010). Tools for consensus analysis of experts’ contours for radiotherapy structure definitions. Radiother. Oncol..

[18] Fedorov A., Beichel R., Kalpathy-Cramer J., Finet J., Fillion-Robin J.C., Pujol S., Bauer C., Jennings D., Fennessy F., Sonka M. (2012). 3D Slicer as an image computing platform for the Quantitative Imaging Network. Magn. Reason. Imaging.

[19] Offersen B.V., Boersma L.J., Kirkove C., Hol S., Aznar M.C., Biete Sola A., Kirova Y.M., Pignol J.P., Remouchamps V., Verhoeven K. (2015). ESTRO consensus guideline on target volume delineation for elective radiation therapy of early stage breast cancer. Radiother. Oncol..

[20] Zeman E.M., Schreiber E.C., Tepper J.E., Niederhuber J.E., Armitage J.O., Kastan M.B., Doroshow J.H., Tepper J.E. (2020). 27—Basics of Radiation Therapy. Abeloff’s Clinical Oncology.

[21] Rucker G., Schimek-Jasch T., Nestle U. (2012). Measuring inter-observer agreement in contour delineation of medical imaging in a dummy run using Fleiss’ kappa. Methods Inf. Med..

[22] Landis J.R., Koch G.G. (1977). The measurement of observer agreement for categorical data. Biometrics.

