# Supplementary material for: Comparison of Dose Distribution in Regional Lymph Nodes in Whole-Breast Radiotherapy vs. Whole-Breast Plus Regional Lymph Node Irradiation: An In Silico Planning Study in Participating Institutions of the Phase III Randomized Trial (KROG 1701)
Source: Cancers (Basel). 2020 Nov 4;12(11):3261. doi: 10.3390/cancers12113261 (PMC7694237; doi:10.3390/cancers12113261)
Supplement: Supplementary file 1 [file cancers-12-03261-s001.pdf]

## Supplementary Material

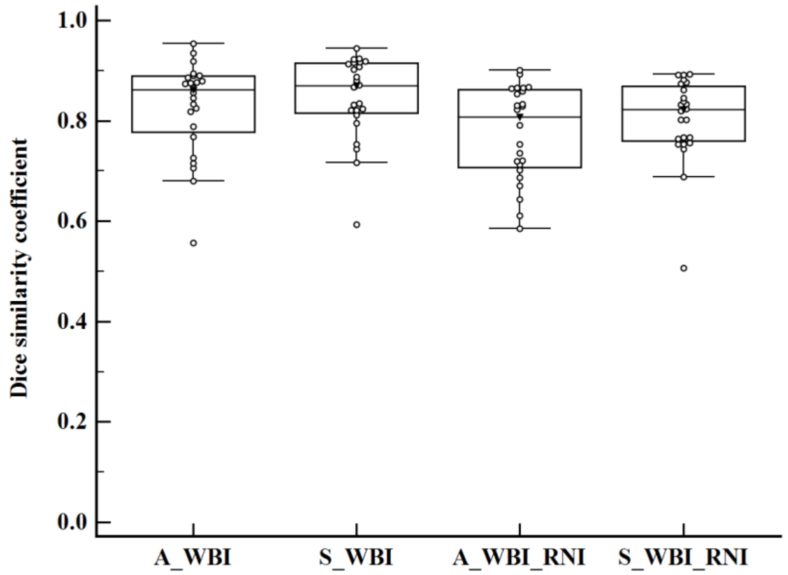


**Figure S1.** Box-and-whisker plots for dice similarity coefficient among 24 participating institutions according to treatment arms. Abbreviations: A WBI, Case A whole breast irradiation; S WBI, Case S whole breast irradiation; A WBI RNI, Case A whole breast irradiation plus regional nodal irradiation; S WBI RNI, Case S whole breast irradiation plus regional nodal irradiation; S WBI RN.

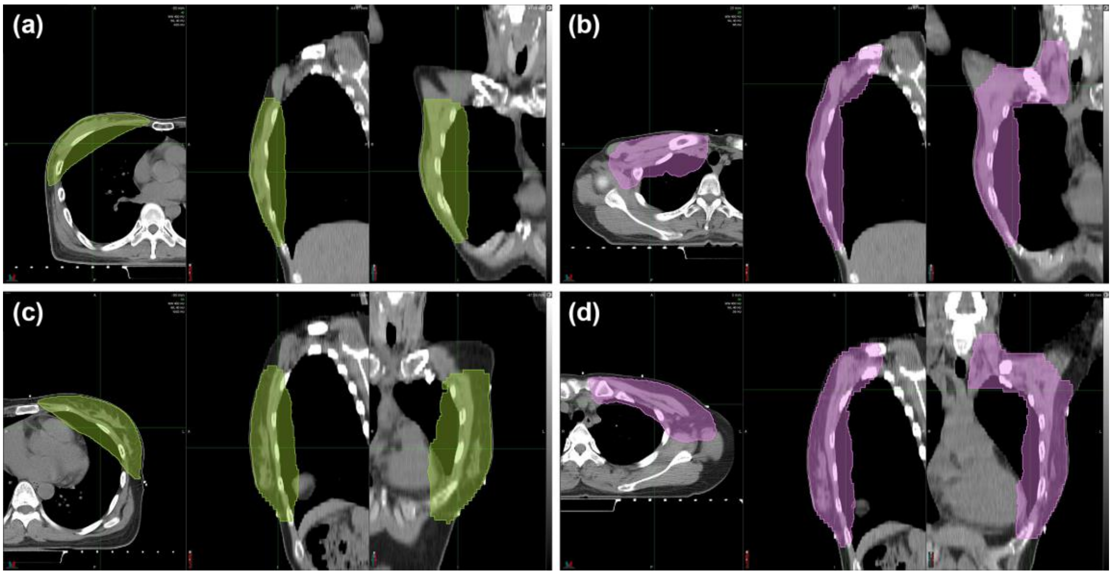


**Figure S2.** A reference 90% isodose contour that was created based on 90% isodose contours of 24 institutions using the STAPLE algorithm. (**a**) WBI arm in Case A, (**b**) WBI+RNI arm in Case A, (**c**) WBI arm in Case S, and (**d**) WBI+RNI arm in Case S. Abbreviations: STAPLE, simultaneous truth and performance level estimation; WBI, whole-breast irradiation; WBI+RNI, whole-breast irradiation plus regional nodal irradiation.

**Table S1.** Relative nodal radiation dose according to the internal mammary lymph node irradiation among WBI+RNI arm.

| Nodal sites | Case A (Rt, ALND) | | | Case S (Lt, SLNBx) | | |
|---|---|---|---|---|---|---|
| | IMN RT (–) ($n$ = 18) | IMN RT (+) ($n$ = 6) | $p$-value | IMN RT (–) ($n$ = 18) | IMN RT (+) ($n$ = 6) | $p$-value |
| ALN I | 1.03 (0.68–1.14) | 1.10 (1.05–1.17) | <0.01 | 0.98 (0.52–1.14) | 1.10 (1.02–1.19) | 0.01 |
| ALN II | 1.08 (0.97–1.15) | 1.12 (1.05–1.14) | 0.25 | 1.06 (0.34–1.14) | 1.08 (1.04–1.15) | 0.18 |
| ALN III | 1.12 (0.93–1.18) | 1.12 (1.03–1.16) | 1.00 | 1.11 (0.14–1.22) | 1.14 (1.03–1.18) | 0.36 |
| SCN | 1.13 (0.69–1.20) | 1.11 (1.06–1.14) | 0.28 | 1.11 (0.18–1.24) | 1.12 (1.06–1.15) | 0.68 |
| IMN | 0.71 (0.07–1.02) | 1.04 (0.94–1.15) | <0.01 | 0.67 (0.29–0.88) | 1.04 (0.87–1.18) | <0.01 |

The median relative radiation among 24 institutions was presented according to nodal groups. The relative nodal dose is defined as the ratio of nodal dose to the target prescription dose. Value in parentheses presents a range of relative nodal dose for each nodal group. Case A represents a patient with right breast cancer who received axillary lymph node dissection. Case S is a patient with left breast cancer who underwent sentinel lymph node biopsy. The *p*-value was calculated using Wilcoxon signed-rank test. Abbreviations: WBI+RNI, whole-breast irradiation plus regional lymph node irradiation; Rt, right; ALND, axillary lymph node dissection; Lt, left; SLNBx, sentinel lymph node biopsy; IMN RT, internal mammary lymph node radiotherapy, ALN, axillary lymph node; SCN, supraclavicular lymph node; IMN, internal mammary lymph node.

**Table S2.** Radiation dose to lung and heart depending on internal mammary lymph node irradiation in WBI+RNI arm.

| Organs | Case A (Rt, ALND) | | | Case S (Lt, SLNBx) | | |
|---|---|---|---|---|---|---|
| | IMN RT (–) ($n$ = 18) | IMN RT (+) ($n$ = 6) | $p$-value | IMN RT (–) ($n$ = 18) | IMN RT (+) ($n$ = 6) | $p$-value |
| Ipsilateral lung | | | | | | |
| $V_{5\,Gy}$ (%) | 46.1 (32.1–99.7) | 57.1 (31.6–98.8) | 0.07 | 46.1 (37.4–93.8) | 62.8 (27.8–99.9) | 0.08 |
| $V_{10\,Gy}$ (%) | 34.4 (19.5–97.4) | 40.8 (19.2–97.4) | 0.28 | 35.6 (25.5–60.1) | 44.9 (17.6–99.9) | 0.38 |
| $V_{20\,Gy}$ (%) | 28.1 (9.6–34.2) | 24.9 (7.0–76.5) | 0.73 | 28.9 (12.6–38.9) | 28.1 (7.2–74.0) | 0.59 |
| $V_{30\,Gy}$ (%) | 22.1 (4.2–30.3) | 14.2 (1.4–38.4) | 0.14 | 21.2 (6.1–34.9) | 16.4 (2.7–48.6) | 0.16 |
| Heart | | | | | | |
| Mean (Gy) | 0.5 (0.2–5.0) | 4.7 (1.4–6.8) | <0.01 | 3.7 (1.5–6.5) | 8.6 (1.8–10.6) | 0.06 |

Value in parentheses presents the range of the value in each parameter. $V_{x\,Gy}$ indicates the percentage of lung volumes receiving more than x Gy. Case A represents a patient with right breast cancer who received axillary lymph node dissection. Case S is a patient with left breast cancer who underwent sentinel lymph node biopsy. The *p*-value was calculated using Wilcoxon signed-rank test. Abbreviations: Rt, right; ALND, axillary lymph node dissection; Lt, left; SLNBx, sentinel lymph node biopsy; WBI, whole-breast irradiation; WBI+RNI, whole-breast irradiation plus regional nodal irradiation.

**Table S3.** Guidelines for radiation treatment in the KROG 1701 study.

| Category | | Guidelines |
|---|---|---|
| Target volume | Whole breast | Cranial border: upper border of palpable breast tissue or inferior border of the clavicular head |
| | | Caudal border: the lowest part of palpable or visible breast tissue |
| | | Medial border: lateral edge of the medial perforating mammary vessel or the edge of the sternum |
| | | Lateral border: lateral breast fold or anterior to the lateral thoracic artery |
| | Regional lymph nodes | Delineate ALN, SCN and/or IMN according to the EORTC contouring guideline [12]. Whether to include the IMN as a target volume is determined according to each institutional policy. |
| Radiation field design | Whole breast field | Individualized modification of fields for adequate covering of whole breast target. |
| | Regional nodal field | Include ALN, SCN, and- or IMN. Field designing depends on the policy of each institution. |
| Dose prescription | Whole breast | Total dose of 45–50.4 Gy at 1.8–2.0 Gy per fraction, or total dose of 39–43.2 Gy at 2.5–3.0 Gy per fraction. Daily treatment, 5 days a week. Tumor bed boost is allowed. |
| | Regional lymph node | Total dose of 45–50.4 Gy at 1.8–2.0 Gy per fraction, or total dose of 39–43.2 Gy at 2.5–3.0 Gy per fraction. Daily treatment, 5 days a week. |
| Radiation techniques | | Three-dimensional conformal radiotherapy. Intensity-modulated radiotherapy is allowed. |

Abbreviations: ALN, axillary lymph node; SCN, supraclavicular lymph node; IMN, internal mammary lymph node.
